# 

**Published:** 1843-08-05

**Authors:** 


### CONTENTS.

Fatal case of Perforation of the Duodenum. By J. W. Ash, M. D. - 169

*Bibliographical Notices.*

Austria; Its Literary, Scientific, and Medical Institutions, with Notes upon the present state of Science, and Guide to the Hospitals and Sanatory Establishments of Vienna. By W. R. Wilde, M. R. I. A., &c. - - 170

Pathological and Surgical Observations on the Diseases of the Joints. By Sir B. C. Brodie, Bart., F. R. S., Seargeant-Surgeon to the King, &c., 174

A Treatise on Protracted Indigestion and its consequences; being the application to the Practical Department of Medicine of the results of an Inquiry into the Laws of the Vital Functions. &c. &c. By A. P. W. Philip, M. D., F. R. S. - - 175

Practical Remarks on Gout, Rheumatic Fever, and Chronic Rheumatism of the Joints, &c. By Dr. Todd. - 175

Wenzel the Oculist, - - - 175

*Retrospect of the Medical Sciences.*

Case of Popliteal Aneurism cured by Compression of the Femoral Artery. By Edward Hutton, M. D., Surgeon to the Richmond Hospital, - - 176

Dr. Willis on Mental Derangement, - 176

Black Cataract, - - - - - 177

Statistics of Anal Fistula, - - 177

Conservation of Mistura Ferri Composita, - - - - - - 177

Iodurated Syrup of Sarsaparilla, - 177

Quinine in Traumatic Fever, - - 178

Pathology and treatment of Rickets and Mollities Ossium, - - - 178

On the Characters and Structural Peculiarities of a group of Morbid Growths in which cancerous affections are included. By Dr. Hodgkin, - - 178

New limits for the period of Lactation, 179

Diagnostic sign of Dislocation of the Thigh-bone into the Ischiatic Notch, 179

Urea discovered in the Blood during the existence of Pulmonic Inflammation, - - - - - - - - 179

Statistics of Cancer, - - - 179

Influence of the Seasons on Diseases and Mortality, - - - - - 179

Paralysis of the Bladder cured by the Tincture of Cantharides, - - 180

Anatomy of the Ganglionic Nerves, - 180

Use of elder-bark in Chronic-dropsies, 180

Dr. Frampton on Corrosive Sublimate, 180

Test for Iodine in Mineral Waters, - 180

